# Host transcriptional response to SARS‐CoV‐2 infection in COVID‐19 patients

**DOI:** 10.1002/ctm2.534

**Published:** 2021-09-21

**Authors:** Nitesh Kumar Singh, Surabhi Srivastava, Lamuk Zaveri, Thrilok Chander Bingi, Rajarao Mesipogu, Santosh Kumar V., Namami Gaur, Nikhil Hajirnis, Pratheusa Machha, Sakshi Shambhavi, Shagufta Khan, Mamilla Soujanya, Tulasi Nagabandi, Rakesh K. Mishra, Karthik Bharadwaj Tallapaka, Divya Tej Sowpati

**Affiliations:** ^1^ CSIR‐Centre for Cellular and Molecular Biology (CSIR‐CCMB) Hyderabad India; ^2^ Department of Medicine Gandhi Hospital Hyderabad India; ^3^ Academy of Scientific and Innovative Research (AcSIR) Ghaziabad Uttar Pradesh India

Dear Editor,

COVID‐19 has an extremely variable prognosis, ranging from asymptomatic and mildly affected individuals to severe disease and death. We have investigated the transcriptional changes in 36 COVID‐19 positive Indian patients hospitalized during the first surge (Figure 1A, Table S1, and Supplementary Methods) against 5 COVID‐19 negative samples. RNA was isolated from naso/oropharyngeal swabs for paired end sequencing using the Illumina Nova‐seq 6000. We identified 251 upregulated (220 protein coding) and 9068 downregulated (3252 protein coding) differentially expressed genes (DEGs) (adjusted *p*‐value < 0.05 and absolute log2 fold change > 1) (Figure 1B, Tables S2 and S3). Seven patients were critical and required intensive care unit (ICU) intervention, while 23 were discharged from COVID‐19 ward (W), although no significant differences could be seen in their transcriptional profiles (Figure 1C). The overall transcriptional reduction, irrespective of disease severity (Figure 1C), is well correlated with the phenomenon of fading host cell functionality and prominent viral protein synthesis, and may be associated with interference in host cellular processes and responses.^1^ The results indicate a diverse transcriptomic profile in response to SARS‐CoV‐2, in line with the variable prognosis seen in many COVID‐19 patients. However, we find robust activation of the innate immune response concomitant with a reduction in the gene expression profiles associated with cardiac, muscular, and neurological processes, as well as peripheral neurosensory markers.

**FIGURE 1:**
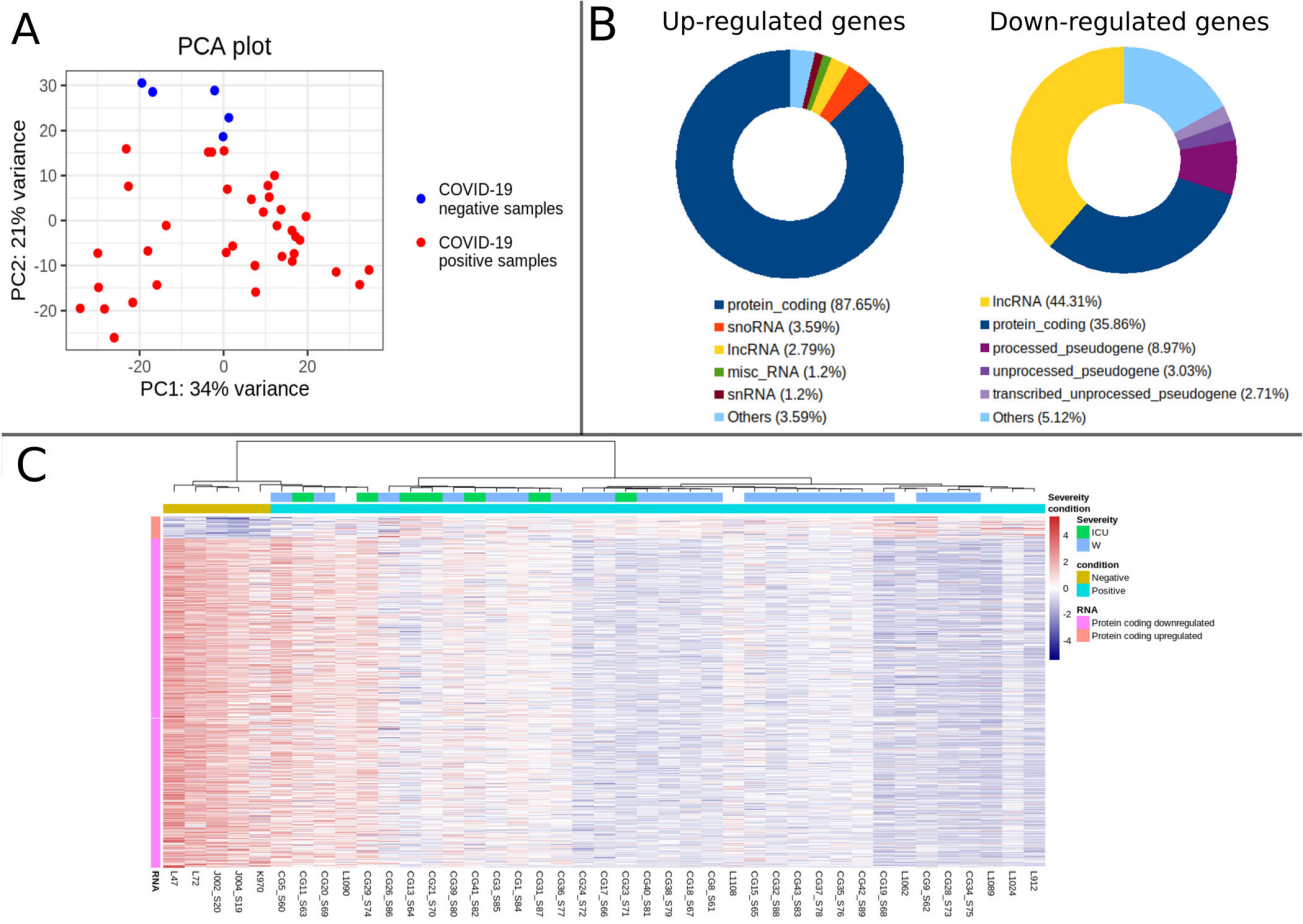
Differentially expressed genes (DEGs) and their expression in COVID‐19 patients compared to controls. (A) Principal component analysis representing the 36 COVID‐19 positive (red) and 5 negative samples (blue). Principal component 1 plotted on the x‐axis explains 34% of the variance and principal component 2 on the y‐axis explains 21% variance in the transcriptomic profiles. (B) Pie chart of biotype composition of DEGs. Only the top five categories for upregulated and downregulated genes are shown. Most of the upregulated genes are protein‐coding but the highest percentage of downregulated genes are lncRNAs. (C) Heatmap of the significantly expressed protein coding genes (rlog transformed expression values). Each bar marks the expression level of a gene from highest (red) to lowest (blue) as per the scale on the right. Sample names are indicated on the x‐axis (clustered by negative and then positive samples) and their metadata is shown on the top. “Condition” indicates COVID‐19 negative (gold bar) or positive (blue bar) status, while “Severity” indicates whether the patients needed ICU intervention (ICU, green bars) or were discharged from the general ward (W, dark blue bars). The Y‐axis bar on the left marks the number of upregulated protein coding genes (orange) followed by the downregulated protein coding genes (pink) in patients compared to the controls

Immune response genes were highly upregulated (Figure 2A and Table S4), with prominent clusters of genes associated with multiple viral infections (Figure 2B and Table S5) marking the activation of infection clearance pathways. Meta‐analysis of published datasets identified a signature of 19 upregulated genes (Table S6), linking Type I interferon (IFN) signaling, host defense, and innate immune responses in SARS‐CoV‐2 infection (Figure 2C). Prominent nodes include well‐documented IFN‐stimulated genes (ISGs), *IFIT* paralogs that restrict viral translation, *IFIH1* and *ISG15* that drive innate immune response upon sensing viral RNA, as well as proviral factors *XAF1* and *MX1*, and DExD/H‐Box Helicase antiviral factors that promote RIG‐I like receptor‐mediated signaling. STAT1 binding to ISGs mediates the IFN‐triggered host response and its dysfunction has been associated with hyperactivation of inflammatory pathways in individuals with acute COVID‐19 pathophysiology. IFN‐mediated activation of the JAK‐STAT signaling pathway may play a role in inducing necroptosis (Figure 2B), and is implicated in Acute Respiratory Distress Syndrome (ARDS) development and protection from severe COVID‐19 along with OAS1.^2^, ^3^ Though not a part of this network, all the MHC class 1 and some MHC class 2 genes (*HLA‐A,B,C,E,F*, and *HLA‐DQB1, DR‐B1, DR‐B5*), involved in T‐cell mediated cell death and the antibody‐mediated adaptive immune response, were also upregulated along with *RFX5*, that binds to MHC‐II promoters. However, many of the proinflammatory markers remained unchanged, suggesting an absence of hyperinflammation and a better disease prognosis in these patients.

**FIGURE 2:**
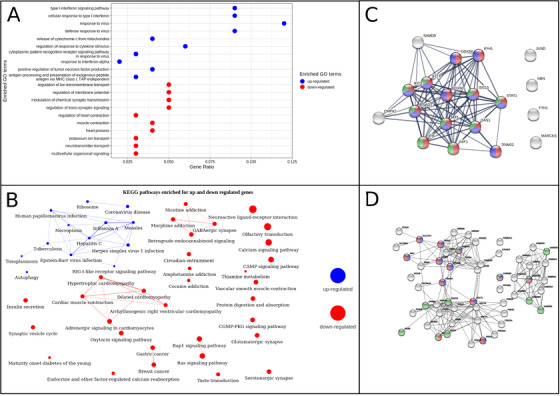
Functional enrichment analysis for protein coding genes (220 upregulated and 3252 downregulated) in COVID‐19 patients compared to controls. (A) Top 10 enriched GO terms associated with upregulated genes (blue) and downregulated genes (red), based on adjusted *p*‐value (p.adj) values. (B) Interaction network showing enriched KEGG pathways associated with upregulated genes (blue nodes) and downregulated genes (red nodes). Nodes sharing 30% or more genes are connected by edges whose thickness represents the percentage of common genes. Size of the node represents the number of genes in that pathway (ranging from 4 to 136). (C) Protein–protein interactions of 19 genes showing a tightly connected network involved in “innate immune response” (red nodes), “defense response to virus” (blue nodes), and “type I interferon signaling pathway” (green nodes). These genes were upregulated during COVID‐19 infection in our analysis as well as in other published datasets (Table S6). Edges depict both functional and physical associations. Edge thickness indicates the confidence in the interaction. All active interaction sources in the STRING database are considered. The minimum interaction score for an edge is set at a high confidence level of 0.7. (D) Protein–protein interactions of 55 genes showing tightly connected clusters of genes involved in “cognition” (red nodes), “learning and memory” (blue nodes), and “sensory perception” (green nodes). These genes were involved in four addiction‐related pathways. Edges depict both functional and physical associations. Edge thickness indicates the confidence in the interaction. All active interaction sources in the STRING database are considered. The minimum interaction score for an edge is set at a high confidence level of 0.7

Downregulated protein coding genes were associated with processes related to neurotransmission and cardiac and muscular contraction (Figure 2A and Table S7). Multiple cardiomyopathy pathways appear to be affected (Figure 2B and Table S8), either as a direct result of the infection or as a downstream consequence of the immune response activation, that may shed light on adverse clinical outcomes. RAS and cAMP signaling pathways, CACNs related to cellular calcium signaling, and key cardiac proteins, such as troponin and tropomyosin, which together with calcium ions are required for proper cardiac muscle contraction, were also downregulated (Figure 2B). These results suggest myocardial issues and highlight the importance of continued follow‐up in COVID‐19 patients.^4^ Pancreatic and insulin secretory systems‐related genes were also downregulated, in agreement with recent work showing that the insulin requirement for patients with diabetes mellitus increases at the peak of COVID‐19 illness.^5^

Interestingly, there was a strong enrichment for “olfactory transduction” and “taste transduction” pathways among downregulated genes (Figure 2B), including 105 olfactory receptor genes. Over the last year, olfactory dysfunction has emerged as a key symptom of COVID‐19 and the loss of smell and taste is likely a consequence of the observed impairment of neurosensory perception pathways.^6^ Genes associated with drug addiction and neuroactive ligand‐receptor pathways also lost their expression pattern. Protein–protein interaction analysis showed two strong networks of genes from the family of gamma‐aminobutyric acid type A (GABA) receptors (Figure 2D), important for normal neurological functioning, and the GRIN genes which are part of the N‐methyl‐D‐aspartate receptors family involved in memory, learning, and synaptic development. Reduction in GABA and alterations in GABA receptor levels are associated with stress‐induced anxiety and depression, increasingly recognized in COVID‐19 patients. The effect on GABAergic interneurons in the olfactory bulb, connecting sensory neurons in the olfactory epithelium, might increase the potential for neurological complications observed in COVID‐19 patients.^7^ Further studies are underway to delineate the implications for neuronal infectivity via the olfactory and respiratory tracts and the nasopharyngeal compartment,^6^ which are predominantly epithelial cells.

A large proportion of the DEGs included relatively low expression lncRNAs (Figure 3A), including some known to have functional roles during viral infection. For example, *ZBTB11‐AS1*, an antisense lncRNA to *ZBTB11*, regulating neutrophil development^8^ was upregulated along with the cognate gene. *HEIH*, associated with recurrence in hepatitis C virus‐related hepatocellular carcinoma and *IGF2‐AS*, associated with HepC viral replication^9^, ^10^ were also significantly misregulated. However, the role of many lncRNAs is unspecified. We identified 720 differentially expressed protein coding genes nearest to the misregulated lncRNAs, most of which were found to overlap the cognate gene or its promoter on the antisense strand (Figure 3B) and potentially mediated many developmentally regulated processes (Figure 3C and Table S9).

**FIGURE 3:**
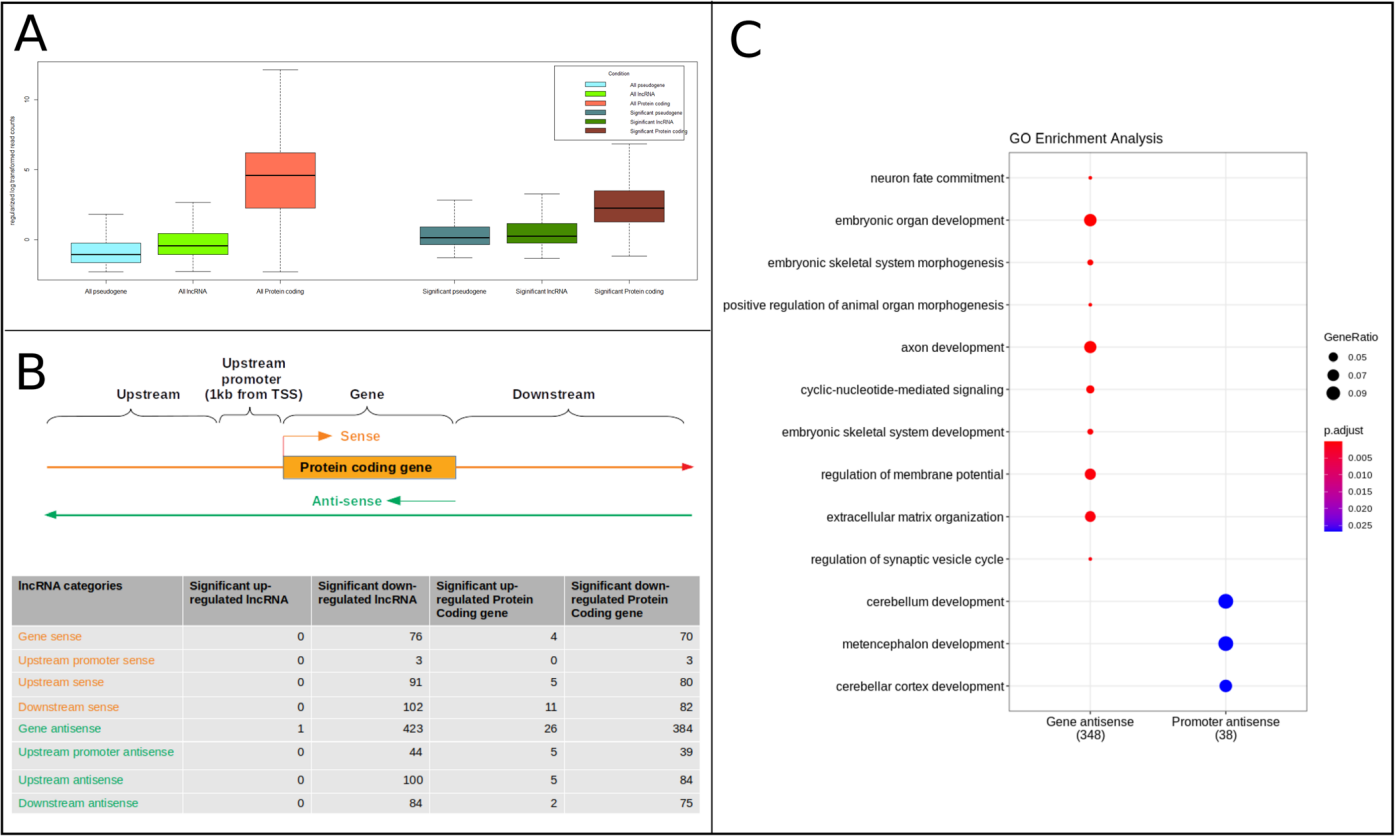
Distribution of expression data and enrichment analysis of differentially expressed protein coding genes closest to lncRNA. (A) Distribution of expression value (rlog transformed) for pseudogenes, lncRNAs, and protein‐coding genes displayed in the form of boxplot. All the pseudogenes, lncRNAs, and protein‐coding genes are shown on the left side of the plot, while differentially expressed genes are shown on the right side of the plot. Median of the distribution is represented by a horizontal line and whiskers are extended to 1.5 times interquartile range. (B) Schematic showing the method employed to categorize differentially expressed lncRNAs. The lncRNAs are divided into eight groups based on their location––genomic strand and distance to their closest differentially expressed protein coding gene. Number of lncRNA and their closest protein coding genes for each category is shown in the table. Most of the lncRNAs were on the antisense strand and overlapped with the cognate gene or its promoter. (C) GO enrichment analysis of the nearest protein coding genes associated with the differentially expressed lncRNAs. Enriched terms were identified only when the associated lncRNA overlapped with the cognate gene or its promoters. Only top 10 enriched terms, based on adjusted *p*‐value (p.adj) values, are plotted. Size of dots represents the number of genes and color signifies p.adj value

In conclusion, we have documented significantly misregulated genes and associated pathways during SARS‐CoV‐2 infection in Indian patients (summarized in Figure 4). Our results highlight a commonly upregulated network of innate immune response genes and absence of hyperinflammatory markers. A majority of the genes being downregulated suggest host shutdown and large‐scale systemic effects spanning not just lung and respiratory complications but also cardiac, endocrine, and neurological issues. The downregulation of a large proportion of sensory receptors, including olfactory and taste receptors, and associated pathways stands out as a major correlate of SARS‐CoV‐2 infection. Such studies can help compare host responses in the current and subsequent waves of the pandemic across the globe and identify targets for monitoring and planning therapeutic approaches.

**FIGURE 4:**
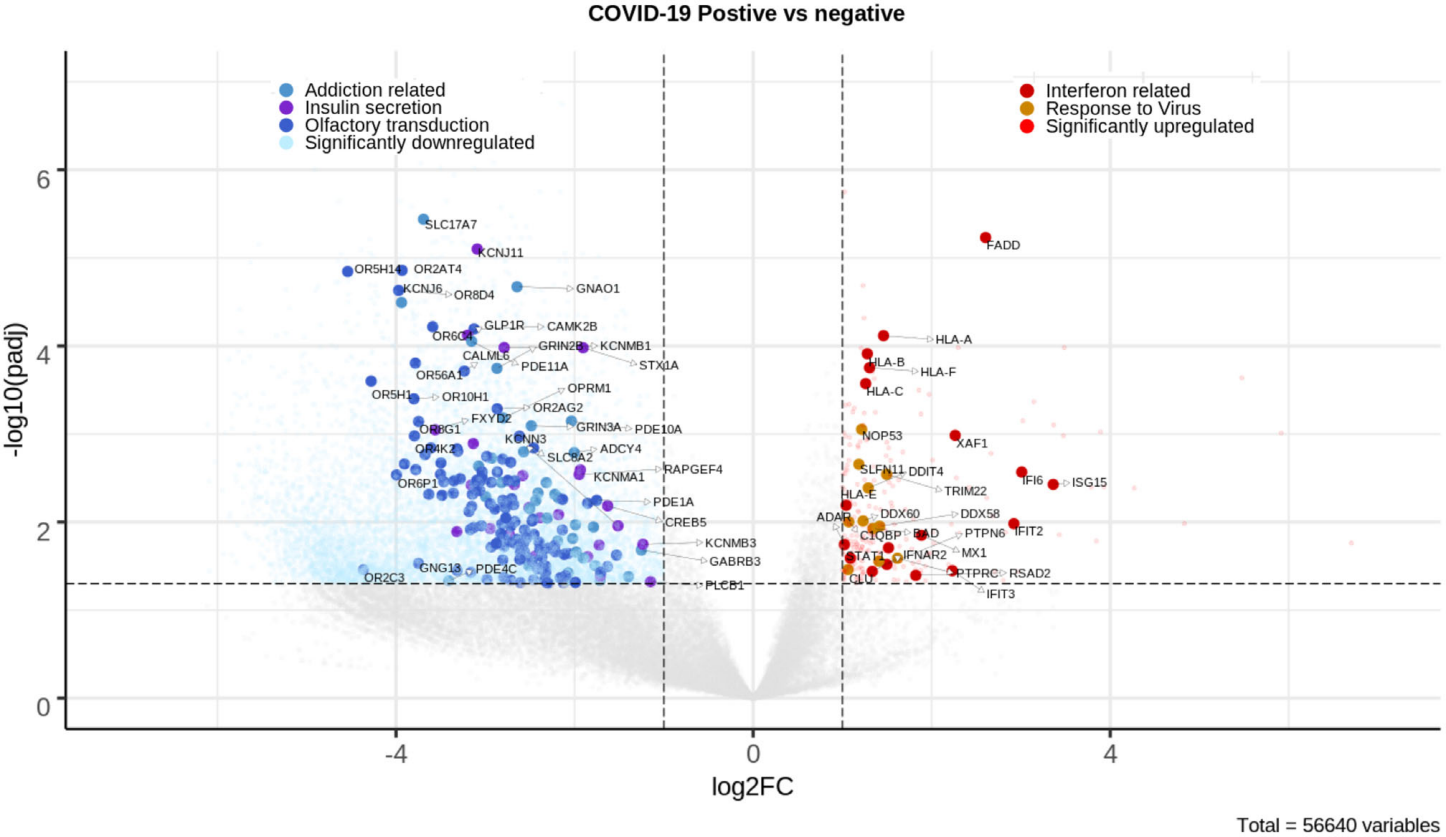
Enriched GO processes and KEGG pathways associated with significant DEGs. Volcano plot showing rlog transformed expression values of all the genes. Each gene is plotted based on the log2 fold change value (X‐axis) and –log10 adjusted *p*‐value (Y‐axis). Vertical dashed line (absolute (log2 fold change) = 1) and horizontal dashed line (padj = 0.05) show the criteria set for defining significant DEGs. Genes not changing significantly are colored gray. Upregulated genes (colored red) are on the right side of the plot, while downregulated genes (colored blue) are on the left side of the plot. Genes from significantly enriched GO terms and KEGG pathways are highlighted. For example, genes labeled as “Interferon related” are associated with the GO terms “type I interferon signaling pathway” and “cellular response to type I interferon.” Genes labeled as “Response to virus” are associated with the GO terms “defense response to virus” and “response to virus.” Genes marked as “Addiction related” are from four enriched KEGG pathways, namely, “Nicotine addiction,” “Morphine addiction,” “Cocaine addiction,” and “Amphetamine addiction.” The other labels correspond to genes from the KEGG pathway “Insulin secretion” and “Olfactory transduction”

## CONFLICT OF INTEREST

The authors declare no conflict of interest.

## Supporting information

Supporting Information

Table S1 : Sample metadata.
Table S2 : Differential expression analysis result from DESeq2 when COVID‐19 positive samples were compared with control.
Table S3 : Biotype composition table for differentially expressed genes.
Table S4 : GO enrichment table for the protein coding genes that were upregulated in COVID‐19 samples.
Table S5 : KEGG enrichment table for the protein coding genes that were upregulated in COVID‐19 samples.
Table S6 : List of genes identified from meta analysis of existing transcriptomic datasets for COVID‐19 samples.
Table S7 : GO enrichment table for the protein coding genes that were downregulated in COVID‐19 samples.
Table S8 : KEGG enrichment table for the protein coding genes that were downregulated in COVID‐19 samples.
Table S9 : GO enrichment analysis of the differentially expressed protein‐coding genes nearest to differentially expressed lncRNAs.

## Data Availability

Raw data and the RNA‐seq count data can be accessed from Gene Expression Omnibus (GEO) database (accession number GSE166530).

## References

[1] Rivas H, Schmaling S, Gaglia M. Shutoff of host gene expression in influenza A virus and herpesviruses: Similar mechanisms and common themes. Viruses. 2016;8(4):102. 10.3390/v8040102 27092522 PMC4848596

[2] Zhou S, Butler‐Laporte G, Nakanishi T et al. A Neanderthal OAS1 isoform protects individuals of European ancestry against COVID‐19 susceptibility and severity. Nature Med. 2021;27(4):659–667. 10.1038/s41591-021-01281-1 33633408

[3] Matsuyama T, Kubli SP, Yoshinaga SK, Pfeffer K, Mak TW. An aberrant STAT pathway is central to COVID‐19. Cell Death Diff. 2020;27(12):3209–3225. 10.1038/s41418-020-00633-7 PMC7545020 33037393

[4] Bader F, Manla Y, Atallah B, Starling RC. Heart failure and COVID‐19. Heart Fail Rev. 2021;26(1):1–10. 10.1007/s10741-020-10008-2 32720082 PMC7383122

[5] Wu L, Girgis CM, Cheung NW. COVID‐19 and diabetes: Insulin requirements parallel illness severity in critically unwell patients. Cli Endocrinol. 2020;93(4):390–393. 10.1111/cen.14288. PMC7404426 32683745

[6] Butowt R, von Bartheld CS. Anosmia in COVID‐19: Underlying Mechanisms and Assessment of an Olfactory Route to Brain Infection. Neuroscientist. 2020;107385842095690. 10.1177/1073858420956905 PMC7488171 32914699

[7] Ellul MA, Benjamin L, Singh B, et al. Neurological associations of COVID‐19. Lancet Neurol. 2020;19(9):767–783. 10.1016/s1474-4422(20)30221-0 32622375 PMC7332267

[8] Keightley M‐C, Carradice DP, Layton JE, et al. The Pu.1 target gene Zbtb11 regulates neutrophil development through its integrase‐like HHCC zinc finger. Nat Commun. 2017;8(1). 10.1038/ncomms14911. PMC5384227 28382966

[9] Xiong Y, Jia M, Yuan J, et al. STAT3‐regulated long non‐coding RNAs lnc‐7SK and lnc‐IGF2‐AS promote hepatitis C virus replication. Mol Med Rep. 2015;12(5):6738–6744. 10.3892/mmr.2015.4278 26328522 PMC4626162

[10] El Samaloty NM, Shabayek MI, Ghait RS, El‐Maraghy SA, Rizk SM, El‐Sawalhi MM. Assessment of lncRNA GAS5, lncRNA HEIH, lncRNA BISPR and its mRNA BST2 as serum innovative non‐invasive biomarkers: Recent insights into Egyptian patients with hepatitis C virus type 4. World J Gastroenterol. 2020;26 (2):168–183. 10.3748/wjg.v26.i2.168 31988583 PMC6962433

