# Supplementary material for: Host transcriptional response to SARS‐CoV‐2 infection in COVID‐19 patients
Source: Clin Transl Med. 2021 Sep 21;11(9):e534. doi: 10.1002/ctm2.534 (PMC8453261; doi:10.1002/ctm2.534)
Supplement: Supplementary file 1 — Supporting Information [file CTM2-11-e534-s002.docx]

**Host transcriptional response to SARS-CoV-2 infection in COVID-19 patients**

Nitesh Kumar Singh^#,1^, Surabhi Srivastava^#,1^, Lamuk Zaveri^#,1^, Thrilok Chander Bingi^2^, Rajarao Mesipogu^2^, Santosh Kumar V^1^, Namami Gaur^1^, Nikhil Hajirnis^1^, Pratheusa Machha^1,3^, Sakshi Shambhavi^1,3^, Shagufta Khan^1^, Mamilla Soujanya^1,3^, Tulasi Nagabandi^1^, Rakesh K. Mishra^1^, Karthik Bharadwaj Tallapaka^1^, Divya Tej Sowpati^1^

^1^ CSIR-Centre for Cellular and Molecular Biology (CSIR-CCMB), Uppal Road, Hyderabad 500007, India.

^2^ Department of Medicine, Gandhi Hospital, Hyderabad

^3^Academy of Scientific and Innovative Research (AcSIR), Ghaziabad, Uttar Pradesh

^#^Equal contribution of authors

**Keywords:** COVID-19, host transcriptome, SARS-CoV-2, innate immunity, olfactory receptors

**Running Title:** Host transcriptional response in COVID19

**Correspondence**: Divya Tej Sowpati, CSIR Centre for Cellular and Molecular Biology (CSIR-CCMB), Uppal Road, Hyderabad 500007, India. [tej@ccmb.res.in](mailto:tej@ccmb.res.in)

# Supplementary Information

# **Methods**

# **Sample metadata**

We analyzed transcriptome profiles from 41 patient samples; 36 samples were confirmed to be COVID-19 positive using RT-PCR method, and five were negative. The age of the patients ranged from 10 to 80 years old. There were 11 females and 30 males in our dataset. We also had severity of COVID-19 for 30 positive patients; 7 patients required intensive care unit (ICU) intervention while 23 were discharged from COVID-19 ward (W). The detailed metadata information about the samples is provided in Supplementary table 1.

## **Sample preparation and sequencing**

This study was approved by the Institutional Ethics Committee to use patient samples for sequencing and all guidelines were followed. The samples were collected from nasopharyngeal or oropharyngeal swabs as previously described [1]. Total RNA was extracted from 1ml of VTM using TRIzol reagent (Thermo Fisher Scientific, USA) or TRIzol in combination with Direct-zol RNA Microprep Kits (Zymo, USA) according to the manufacturer’s protocol. RNA was quantified using Qubit RNA HS Assay Kit (Thermo Fisher Scientific, USA) and 1 µg total RNA was used for library preparation. Ribosomal RNA was removed using the RiboCop rRNA Depletion Kit (Lexogen, Austria).

RNA-Seq libraries were made using the CORALL Total RNA-Seq Library Prep Kit (Lexogen, Austria) according to the manufacturer’s protocol. Briefly, rRNA depleted RNA was reverse transcribed using displacement stop primers which contain partial Illumina-compatible P7 sequences. Linkers containing partial Illumina-compatible P5 sequences and Unique Molecular Identifiers were ligated to the 3’ end of cDNA fragments. The library was PCR amplified to add the remaining adapter sequences and 12 nucleotide unique indices for multiplexing. Samples were sequenced at PE150 using the Illumina Nova Seq 6000.

**Data Processing and Analysis**

Raw sequencing reads were checked for quality using FASTQC v0.11.9 [2] and Illumina universal adapters and low-quality reads were removed using cutadapt 2.8 [3]. We removed reads with quality scores less than 20 and discarded reads smaller than 36 bp. The processed reads were then again checked for quality using FASTQC and then mapped to the human genome GRCh38 using STAR 2.7.3a with default parameters [4]. The STAR index was generated using the GRCh38 genome and annotation gtf file downloaded from ENSEMBL [5]. BAM files generated were then sorted according to position using Samtools 1.10 [6]. The quality of BAM was assessed using QualiMap v.2.2.2-dev [7]. All the quality reports were compiled using multiqc 1.9 [8]. Uniquely aligned reads were counted using HTSeq [9]. There were 60683 genes in the gtf file for which we had count information. Genes with read count 0 across all the samples were removed resulting in 56640 genes for further analysis. Differential gene expression analysis was performed using DESeq2_1.24.0 [10]. Genes with adjusted p-value < 0.05 and absolute log2 Fold change > 1 were considered differentially expressed resulting in 9319 genes, remaining 47321 genes were removed from further analysis. For PCA plot and heat map, the raw read counts were rlog normalized, available with the DESeq2 package.

**Functional enrichment analysis**

For functional enrichment analysis, clusterProfiler_3.12.0 [11] was used for GO term enrichment and ShinyGO [12] was used for Kyoto Encyclopedia of Genes and Genomes (KEGG) pathway analysis. We only used the Biological process for GO term enrichment analysis. Similar enriched terms were further merged using the ‘simplify’ function of clusterProfiler with similarity cutoff set to 0.7. ‘p.adjust’ was used as a feature to select representative terms and ‘min’ was used to select features. ‘Wang’ was used as a method to measure similarity. For ShinyGO the web tool was run with the default parameter. The complete list of enriched KEGG pathways were downloaded. For better clarity, only the top 20 significant pathways based on FDR were shown in the figures. Edge cut-off was set to 0.3, so edges were created only if two nodes shared at least 30% of the genes. Darker nodes represent more significantly enriched pathways and size of the node represents the number of input genes in that pathway. Thickness of edges was proportional to percentage of overlapping genes.

**Protein-protein interaction network**

Protein-protein interaction network was created using STRING-db [13]. Both physical and functional protein associations were considered for edges. All the active interaction sources were considered for generating the network. The medium confidence required for interaction score was set to 0.7. Only input proteins were used for generating the network. Few chosen enriched biological processes or KEGG pathways as reported by STRING-db were shown by color code of the nodes.

**Meta-analysis of existing transcriptomic data for COVID-19**

We performed data mining and compiled a list of 9 published works on transcriptomic host response to COVID-19 [14–22]. We then overlapped our up-regulated/down-regulated genes with a list of genes that were reported to be up-regulated/ down-regulated in the published datasets. For down-regulated genes the overlap was not good between the published dataset. For up-regulated genes, we generated a list of genes that were found to be up-regulated in at least 3 publications. These 19 genes are given in Supplementary table 6.

**Identifying nearest genes for the lncRNA**

We used bedtools (v2.26.0) [23] to find nearest genes for the lncRNA. Bedtools closest searches for overlapping features in two bed files. If there is no overlap, the nearest feature is reported with genomic distance between the two features. Closest features were reported irrespective to strand. The bed file containing differentially expressed lncRNA genomic locations was used as a query bed file (option -a) and a bed file containing all protein coding RNA genomic locations was used as reference bed file (option -b). The lncRNA were classified based on the closest gene. If both the gene and lncRNA were on the same strand, lncRNA was classified as “sense”, otherwise it was classified as “anti-sense”. Next, the lncRNA was classified based on the distance from the closest gene. If the gene and lncRNA were overlapping, lncRNA was classified as “Gene”. If the lncRNA is within 1kb upstream from the closest gene, it is classified as “upstream promoter”. Finally, lncRNA upstream of the closest gene by more than 1 kb was classified as “upstream’ and lncRNA downstream of the gene was classified as “downstream”. This resulted in 8 sets of classification for the lncRNA (Figure S2A). Further, Gene Ontology analysis showed enrichment of terms only in the groups where the lncRNA was located within the gene or in its upstream promoter (Figure S2B).

**References**

1. Banu S, Jolly B, Mukherjee P, et al (2020) A Distinct Phylogenetic Cluster of Indian Severe Acute Respiratory Syndrome Coronavirus 2 Isolates. Open Forum Infectious Diseases. https://doi.org/10.1093/ofid/ofaa434

2. Andrews S (2010) FastQC - A quality control tool for high throughput sequence data. http://www.bioinformatics.babraham.ac.uk/projects/fastqc/. Babraham Bioinformatics

3. Martin M (2011) Cutadapt removes adapter sequences from high-throughput sequencing reads. EMBnet.journal. https://doi.org/10.14806/ej.17.1.200

4. Dobin A, Davis CA, Schlesinger F, et al (2013) STAR: Ultrafast universal RNA-seq aligner. Bioinformatics. https://doi.org/10.1093/bioinformatics/bts635

5. Yates AD, Achuthan P, Akanni W, et al (2020) Ensembl 2020. Nucleic Acids Research. https://doi.org/10.1093/nar/gkz966

6. Li H, Handsaker B, Wysoker A, et al (2009) The Sequence Alignment/Map format and SAMtools. Bioinformatics. https://doi.org/10.1093/bioinformatics/btp352

7. Okonechnikov K, Conesa A, García-Alcalde F (2016) Qualimap 2: Advanced multi-sample quality control for high-throughput sequencing data. Bioinformatics. https://doi.org/10.1093/bioinformatics/btv566

8. Ewels P, Magnusson M, Lundin S, Käller M (2016) MultiQC: Summarize analysis results for multiple tools and samples in a single report. Bioinformatics. https://doi.org/10.1093/bioinformatics/btw354

9. Anders S, Pyl PT, Huber W (2015) HTSeq-A Python framework to work with high-throughput sequencing data. Bioinformatics. https://doi.org/10.1093/bioinformatics/btu638

10. Love MI, Anders S, Huber W (2014) Differential analysis of count data - the DESeq2 package

11. Yu G, Wang LG, Han Y, He QY (2012) ClusterProfiler: An R package for comparing biological themes among gene clusters. OMICS A Journal of Integrative Biology. https://doi.org/10.1089/omi.2011.0118

12. Ge SX, Jung D, Jung D, Yao R (2020) ShinyGO: A graphical gene-set enrichment tool for animals and plants. Bioinformatics. https://doi.org/10.1093/bioinformatics/btz931

13. Szklarczyk D, Gable AL, Lyon D, et al (2019) STRING v11: Protein-protein association networks with increased coverage, supporting functional discovery in genome-wide experimental datasets. Nucleic Acids Research. https://doi.org/10.1093/nar/gky1131

14. Ng DL, Granados AC, Santos YA, et al (2021) A diagnostic host response biosignature for COVID-19 from RNA profiling of nasal swabs and blood. Science advances 7:eabe5984. https://doi.org/10.1126/sciadv.abe5984

15. Emanuel W, Kirstin M, Vedran F, et al (2020) Bulk and single-cell gene expression profiling of SARS-CoV-2 infected human cell lines identifies molecular targets for therapeutic intervention. bioRxiv. https://doi.org/10.1101/2020.05.05.079194

16. Zhou Z, Ren L, Zhang L, et al (2020) Heightened Innate Immune Responses in the Respiratory Tract of COVID-19 Patients. Cell Host and Microbe. https://doi.org/10.1016/j.chom.2020.04.017

17. Blanco-Melo D, Nilsson-Payant BE, Liu WC, et al (2020) Imbalanced Host Response to SARS-CoV-2 Drives Development of COVID-19. Cell. https://doi.org/10.1016/j.cell.2020.04.026

18. Lee JS, Park S, Jeong HW, et al (2020) Immunophenotyping of covid-19 and influenza highlights the role of type i interferons in development of severe covid-19. Science Immunology. https://doi.org/10.1126/sciimmunol.abd1554

19. Singh K, Chen Y-C, Hassanzadeh S, et al (2021) Network Analysis and Transcriptome Profiling Identify Autophagic and Mitochondrial Dysfunctions in SARS-CoV-2 Infection. Frontiers in Genetics 12:599261. https://doi.org/10.3389/fgene.2021.599261

20. Vishnubalaji R, Shaath H, Alajez NM (2020) Protein coding and long noncoding RNA (lncRNA)) transcriptional landscape in SARS-CoV-2 infected bronchial epithelial cells highlight a role for interferon and inflammatory response. Genes. https://doi.org/10.3390/genes11070760

21. Butler D, Mozsary C, Meydan C, et al (2021) Shotgun transcriptome, spatial omics, and isothermal profiling of SARS-CoV-2 infection reveals unique host responses, viral diversification, and drug interactions. Nature Communications 12:. https://doi.org/10.1038/s41467-021-21361-7

22. Tang H, Gao Y, Li Z, et al (2020) The noncoding and coding transcriptional landscape of the peripheral immune response in patients with COVID‐19. Clinical and Translational Medicine 10:. https://doi.org/10.1002/ctm2.200

23. Quinlan AR, Hall IM (2010) BEDTools: A flexible suite of utilities for comparing genomic features. Bioinformatics. https://doi.org/10.1093/bioinformatics/btq033

24. Gil N, Ulitsky I (2020) Regulation of gene expression by cis-acting long non-coding RNAs. Nature Reviews Genetics 21:102–117
